# Quantifying Golgi structure using EM: combining volume-SEM and stereology for higher throughput

**DOI:** 10.1007/s00418-017-1564-6

**Published:** 2017-04-20

**Authors:** Sophie Ferguson, Anna M. Steyer, Terry M. Mayhew, Yannick Schwab, John Milton Lucocq

**Affiliations:** 1 Structural Cell Biology Group, School of Medicine, University of St Andrews, North Haugh, Fife, KY16 9TF Scotland UK; 2 School of Life Sciences, Queen’s Medical Centre, University of Nottingham, Nottingham, NG7 2UH UK; 3 Cell Biology and Biophysics Unit, European Molecular Biology Laboratory, Meyerhofstrasse 1, 69117 Heidelberg, Germany

**Keywords:** Golgi, Stereology, Volume-SEM, FIBSEM, SBF-SEM, Sampling, Quantification

## Abstract

Investigating organelles such as the Golgi complex depends increasingly on high-throughput quantitative morphological analyses from multiple experimental or genetic conditions. Light microscopy (LM) has been an effective tool for screening but fails to reveal fine details of Golgi structures such as vesicles, tubules and cisternae. Electron microscopy (EM) has sufficient resolution but traditional transmission EM (TEM) methods are slow and inefficient. Newer volume scanning EM (volume-SEM) methods now have the potential to speed up 3D analysis by automated sectioning and imaging. However, they produce large arrays of sections and/or images, which require labour-intensive 3D reconstruction for quantitation on limited cell numbers. Here, we show that the information storage, digital waste and workload involved in using volume-SEM can be reduced substantially using sampling-based stereology. Using the Golgi as an example, we describe how Golgi populations can be sensed quantitatively using single random slices and how accurate quantitative structural data on Golgi organelles of individual cells can be obtained using only 5–10 sections/images taken from a volume-SEM series (thereby sensing population parameters and cell–cell variability). The approach will be useful in techniques such as correlative LM and EM (CLEM) where small samples of cells are treated and where there may be variable responses. For Golgi study, we outline a series of stereological estimators that are suited to these analyses and suggest workflows, which have the potential to enhance the speed and relevance of data acquisition in volume-SEM.

## Introduction

### Electron microscopy (EM): a tool of choice for high-resolution quantitation

The Golgi complex comprises a stack of membrane-bound cisternae associated with tubules and vesicles (Farquhar and Palade 1981, 1998; Berger and Roth 1997) and functions in the transport, processing and sorting of cargo from the endoplasmic reticulum (ER, Farquhar and Hauri 1997; Barlowe 2002; Geva and Schuldiner 2014). Currently, important questions remain about the Golgi’s molecular and structural framework, its mechanisms of cargo transport (Day et al. 2013; Glick and Luini 2010; Papanikou and Glick 2014), cell cycle transformations (Lucocq and Warren 1987; Lucocq et al. 1989, 1995; Lippincott-Schwartz and Liu 2006; Colanzi et al. 2007; Tang and Wang 2013) and the coordinated function of its stack in transport (Day et al. 2013; Lavieu et al. 2013, 2014). Increasingly, answers have been sought by applying high-throughput techniques that can analyse extensive arrays of experimental or genetic manipulations (Simpson et al. 2012; Verissimo and Pepperkok 2013; Flottman et al. 2013). Electron microscopy (EM) is a key tool for answering these questions at a meaningful and informative resolution.

Light microscopy (LM) is well suited to revealing dynamics of Golgi function and can be automated easily, allowing arrays of experimental or genetic conditions to be evaluated. However, LM lacks the resolution needed for visualising the structural elements of the Golgi directly (Durisic et al. 2014). EM is a more suitable tool because it can visualise membranes, tubules, vesicles, (Amos and Grimstone 1968; Lucocq et al. 1995; Trucco et al. 2004; Martínez-Alonso et al. 2013; Beznoussenko et al. 2014; Mourik et al. 2015) as well as fine details such as structural stages in vesicle and cisternal biogenesis (Amos and Grimstone 1968; Lucocq et al. 1995; Pellet et al. 2013; Martínez-Alonso et al. 2013). EM also displays the Golgi in a cellular context allowing simultaneous study of related membrane traffic organelles such as ER and plasma membrane.

### State of play in TEM

**Fig. 1:**
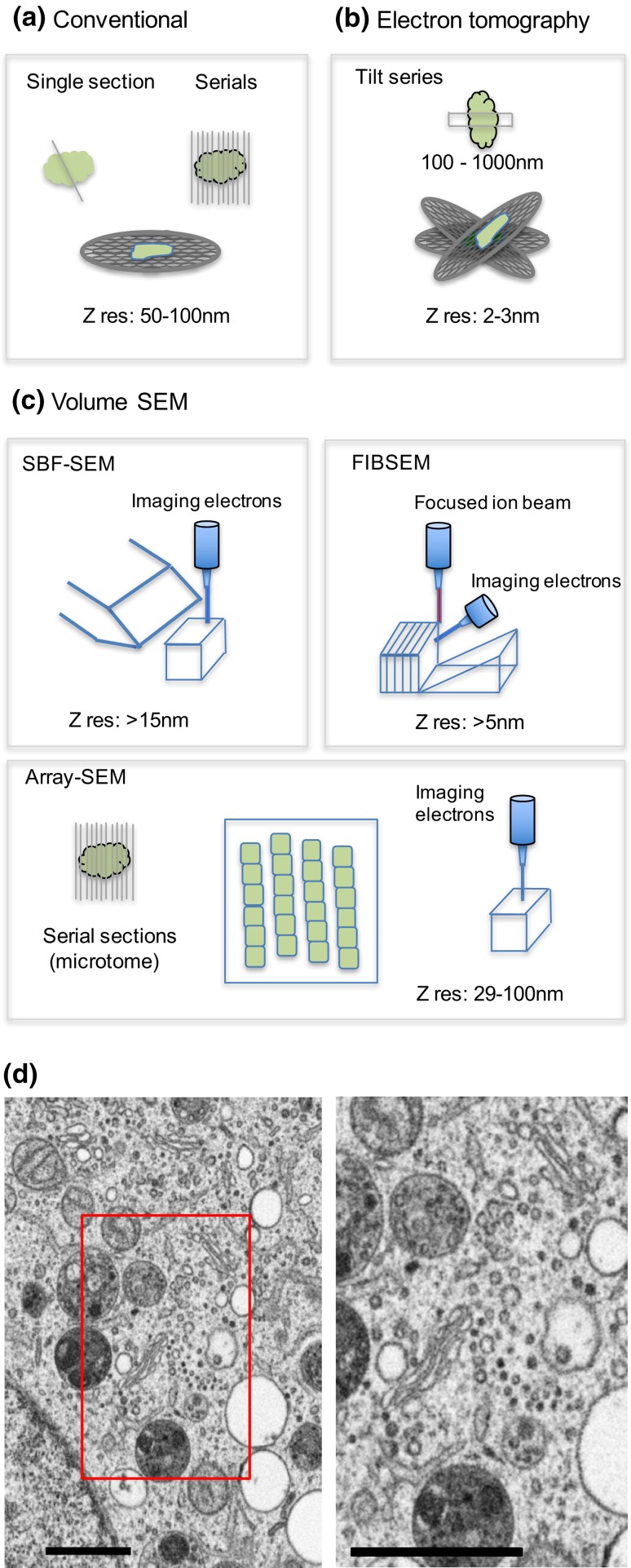
New volume-SEM techniques compared to conventional and tomographic TEM analysis. Using conventional techniques comprehensive 3D information has been acquired using either serial sections (**a** 50-100 nm thickness) or electron tomography (**b** z resolution as little as 2–3 nm). Tomography involves tilting of thicker samples (200-1000nm; Lindsay and Ellisman, 1985; Walther et al. 2013) and back projection to provide 3D information (Donohoe et al. 2006; Marsh and Pavelka 2013; Marsh et al. 2004). However, the usefulness of tomography for whole Golgi studies has been limited due to the comparatively shallow sample depth and narrow scale. These serial section/imaging techniques are labour intensive in obtaining full 3D datasets of Golgi. Volume-SEM techniques (**c**) automate collection of exhaustive serial section/images and include serial block face (SBF-SEM) and focused ion beam SEM (FIBSEM) and array-SEM. SBF-SEM and FIBSEM improve z resolution with section/imaging thickness between 5 and 30 nm and work by iterative removal of sample block-face by physical slicing (SBF-SEM) or by ion beam erosion (FIBSEM), followed by imaging in the SEM. Improved z resolution yields large datasets requiring storage *in silico*, and prompts development and unbiased data mining using stereology. SBF-SEM techniques are adapted to imaging wider scales of tissue (Bosch et al. 2015; Kuwajima et al. 2013; Titze and Genoud 2016), while FIBSEM is narrow-scale allowing focused analysis on single cells. Imaging the block-face improves stability of the 3D array. Array-SEM involves preparation of large arrays of serial sections on conventional microtomes, and section thickness can be little as 29 nm (Kasthuri et al. 2015). Typically, sections are collected on tapes, which are mounted in short series on hard supports prior to SEM imaging. Sections can be prone to dimensional instability and folding. In this case sections can be re-probed reducing the need for *in silico* storage. **d** Illustrates a typical image from of a Golgi complex region of a HeLa cells obtained using FIBSEM imaging (5 nm pixels). *Boxed area* in low magnification image (*left*) is displayed at higher magnification (*right*). *Bars* 1 µM

Transmission EM (TEM) has been a principal tool for quantifying the Golgi for several decades but it has certain disadvantages which include the inefficient acquisition of accurate data, slow speed of specimen preparation (Robinson 1980; Oke and Suarez-Quian 1992; Yelinek et al. 2009) and rather protracted procedures for searching, focussing and image capture. A further problem is the reliance on small and two-dimensional imaging “windows” that are produced when ultrathin sections are examined at high magnifications. The information in these images is often perceived as poorly representing 3D structure or the wider scale quantities of organelles (Lucocq et al. 2015). One possible solution is to acquire 3D data by serial section reconstruction (Fig. 1a; Mogelsvang et al. 2003; Yelinek et al. 2009) or electron tomography (Fig. 1b; Marsh et al. 2001, 2004; Mogelsvang et al. 2003; Marsh and Pavelka 2013). But these techniques are even more labour intensive than conventional TEM and concentrate efforts on limited regions of the sample. Another solution is to sense 3D quantities over a wider scale using stereology (Weibel 1979; Elias and Hyde 1980; Lucocq 1993; Howard and Reed 2005; Nyengaard and Gundersen 2006), a method that utilises random sampling combined with estimation of 3D structural quantities to link 2D quantities with 3D reality. Interestingly, stereology is now becoming more popular (Pubmed search returns for the term “stereology” have increased fivefold in 20 years), but it is still underused in the Golgi field (see Griffiths et al. 1984, 1989; Lucocq et al. 1989, 1995; Bannykh et al. 1996; Seguí-Simarro and Staehelin 2006).

### Volume-SEM techniques: new opportunities for quantifying Golgi

Over the past decade there have been breakthroughs in specimen preparation and imaging for EM, each having the potential to improve the efficiency and throughput of Golgi quantitation. Collectively, these developments are termed volume-SEM (Fig. 1c; Titze and Genoud 2016) and involve successive and automated removal of thin layers or sections of sample, followed by imaging in a high-resolution scanning EM (SEM). The methods generate large series of sections or images that can be wide ranging in area (because of the wide field nature of SEM imaging) and extensive in the direction of sectioning yielding large amounts of 3D information. In all the volume SEM approaches imaging is achieved using high-resolution SEMs with sufficient *x, y* resolution to reveal most Golgi structures although this remains inferior to TEM (Wanner et al. 2013; Hughes et al. 2017; Koga et al. 2016).

Two volume-SEM methods use automated removal of thin layers of specimen inside an SEM chamber followed by automated scanning-electron imaging at high resolution. In serial block face SEM (SBF-SEM; Leighton 1981; Denk and Horstmann 2004; Hughes et al. 2014), a conventional microtome situated inside the microscope chamber shaves thin layers successively from the sample. SBF-SEM produces images that measure millimetres across and have a minimal shaving z-thickness of approximately 30 nm (Titze and Genoud 2016). Even thinner sections are possible under special circumstances (Titze and Genoud 2016), allowing isotropic data assemblies (with voxels of about 10 nm in *x, y* and *z* directions). Initially, the SBF-SEM technique was used to study connectivity in millimetre-sized chunks of brain tissue (Kuwajima et al. 2013), but has been used successfully to image Golgi and other membranous organelles (Hughes et al. 2017). The wide scale view produced by SBF-SEM makes it ideal for sensing large populations of Golgi complexes in tissues or cell cultures although more restricted investigations on individual cells are also possible. The second method uses a focused ion beam to erode the sample in successive layers inside the SEM (FIBSEM; Fig. 1c, d; Ballerini et al. 1997; Knott et al. 2011; Wei et al. 2012; Bosch et al. 2015; Narayan and Subramaniam 2015). Here, block-face size is limited by technical factors to a few tens of microns. Therefore, FIBSEM is suited to analysis of individual cells or groups of cells, rather than the wide-scale imaging of other volume-SEM methods. Importantly, the effective thickness can approach 5 nm, which facilitates isotropic imaging. Typically the specimen is first eroded to form a trench. The end-wall of the trench, which is most often oriented orthogonal to the horizontal, is imaged using SEM. This image plane orientation has implications for the use of quantitative stereological tools (see below).

A third method is best classified as section array-SEM (Fig. 1c; Micheva and Smith 2007; Hayworth et al. 2014; Titze and Genoud 2016; Wacker et al. 2016) and uses the surface imaging capability of SEM on extensive arrays of sections prepared outside the SEM on a conventional TEM microtome. One interesting approach collects sections on a continuous tape substrate before mounting them in arrays on a hard substrate for SEM imaging (Hayworth et al. 2014; Koga et al. 2016). The advantage with array-SEM is that sections can be processed (stained/immunostained) after slicing and re-examined or sampled at any time, greatly reducing image storage needs. Software exists for mapping and imaging in a semi-automated manner (Domart et al. 2012; Hayworth et al. 2014). A major difference from block-face techniques is that the sections in array SEM are thicker, 50–100 nm, although they can be as thin as 29 nm (Kasthuri et al. 2015); and like TEM sections are often prone to section compression and folding.

### Improving efficiency: sampling combined with design-based estimators

The facile generation and imaging of thousands of sections in volume-SEM is a major improvement on TEM, allowing generation of 3D information either by manual segmentation or image recognition software. Models of 3D structure can then be reassembled and probed quantitatively (Tsai et al. 2014). Thus, the temptation in volume-SEM is to store and analyse all the data from the section/image arrays. This approach has advantages because segmentation and 3D reconstruction preserve a rich array of information about position, connectivity and shape of Golgi structures (Hughes et al. 2017). But it is also time consuming and labour intensive and only small numbers of the cells in a population can be examined reliably. This reduces the knowledge of biological or experimental variation and limits relevance of the data to the Golgi population (Lucocq et al. 2015).

The challenge in volume-SEM is how to sense quantitative data more rapidly with less work but at the same time make it relevant to the wider experimental context. One solution is to use a sampling-based stereological approach. Stereological protocols first select tiny fractions of the slices and/or SEM images, prior to quantifying selected structural quantities using appropriate estimators (Shomorony et al. 2015). In this way the careful design of stereology preserves links with the real arrangement of matter in the 3D structure of the biological object (the morphome; Lucocq et al. 2015; Mayhew and Lucocq 2015). This qualifies stereology, along with serial-section reconstructions, as a *bona fide* “morphomics” method (for further discussion, see Lucocq et al. 2015; Mayhew and Lucocq 2015). Significantly, the stereological approach can reduce the amount of work done on individual cells while providing precise and minimally biased estimates of 3D quantities.

**Fig. 2:**
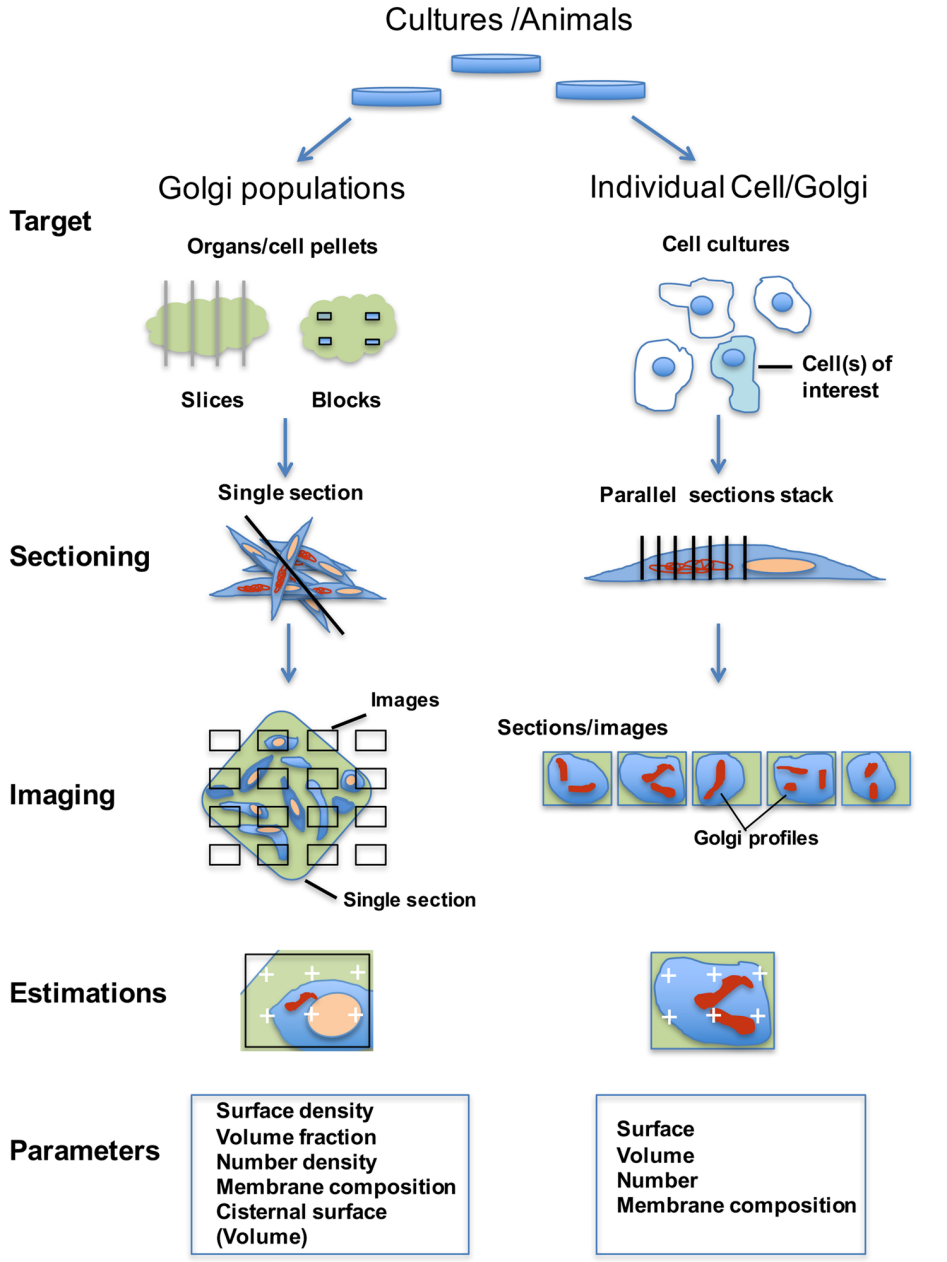
Sampling strategies allowing the use of single sections or parallel section stacks. The population may comprise animals, cell cultures or subcellular Golgi fractions from which organs or cell/organelle pellets or culture dishes are processed for fixation. *Left* Single section analysis on Golgi populations. The items (organs/cell pellets) are fragmented or sliced systematic uniform random (SUR) before being further divided to provide specimen blocks. For single section analysis, blocks are most conveniently sectioned by microtomy (inside the SEM (SBF-SEM) or on a conventional microtome, (array-SEM) before imaging using SEM). Typically, 10–20 images are taken in an SUR array. Estimations are carried out using geometric probes applied to the images (see Figs. 3, 4, 5). *Right* parallel section stacks on Golgi from single cells of interest. Randomly selected dishes and randomly selected cells from subpopulations of interest (e.g. those obtained using CLEM) are identified before, during or after processing for SEM. Golgi complexes of interest are relocated, and a randomly placed section stack with equal spacing is generated through the organelle. Images of the entire Golgi structure are probed using estimators for volume, surface and number on all sections of the stack (Fig. 6). The sampling intensity (5–10 sections) not only provides useful precision on individual cells/Golgi but also increases the number of cells that can be analysed, as compared to exhaustive serial section/image analysis (see text)

The first step in stereology is random sampling, which removes selection bias and preserves reliable links between images and 3D quantities [described in the Fig. 2 (left)]. Random sampling ensures non-preferential selection of items, slice positions and, where necessary, slice orientations through each of the Golgi elements in a population. When random sampling is applied at all levels of sample selection (from animals/cell cultures, specimen blocks, sections, images to estimator probes), the accompanying structural estimates can be unbiased. A modification to random sampling, called systematic uniform random (SUR) sampling, can improve precision further (Fig. 2 left; Gundersen and Jensen 1987; Lucocq 2012). Here samples, sections, images and even estimator probes, are used in a regular array. The SUR sampling array is much easier to apply than random sampling and makes quantitative sensing more efficient when dealing with the heterogeneous structure of biological samples. Extensive experience shows that SUR sampling tends to be more efficient than simple random sampling of biological specimens and decades of research results have confirmed its power in quantitative analysis (Howard and Reed 2005; Mayhew 2008).

**Fig. 3:**
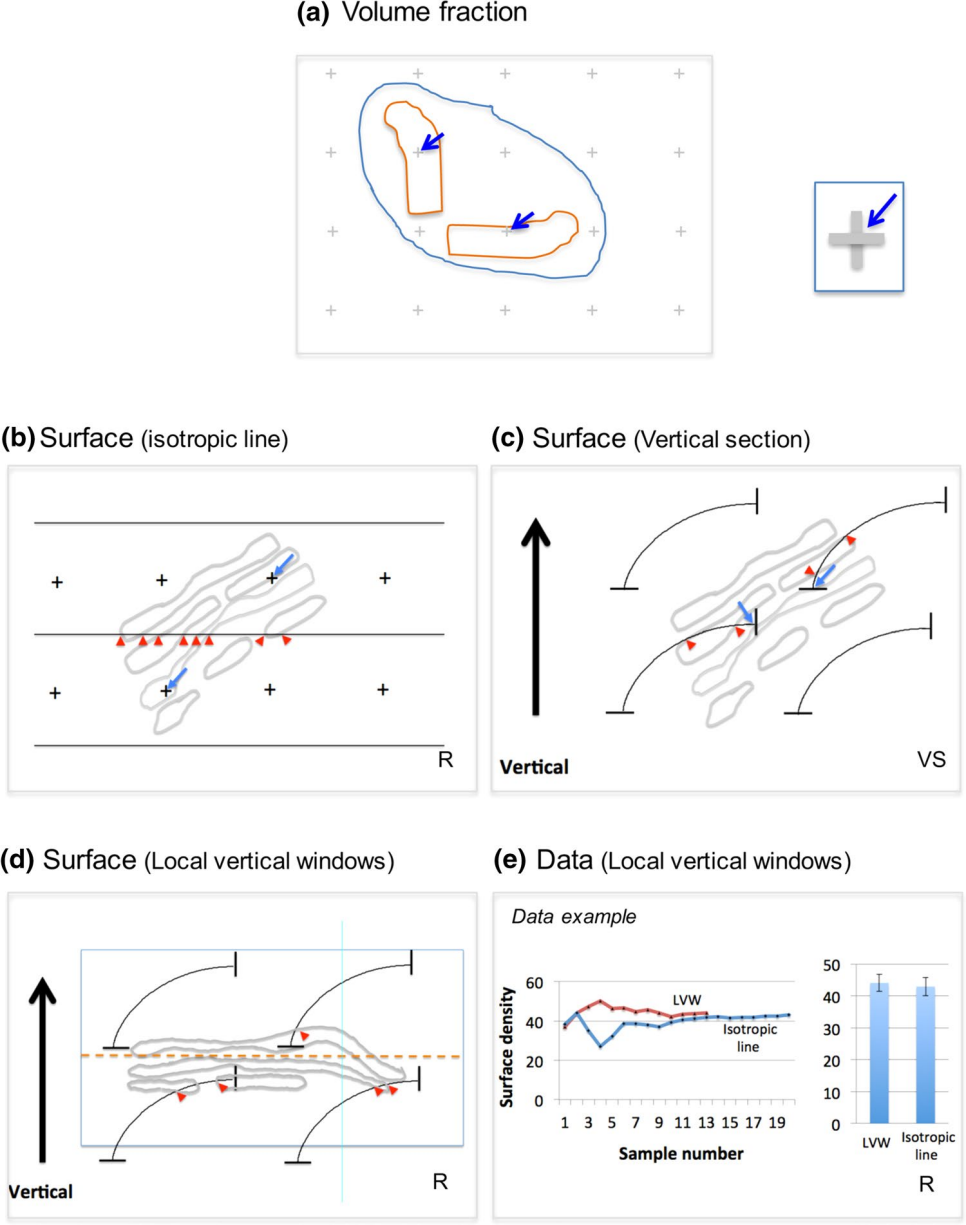
Estimation of volume and surface from single sections. **a** The packing density of structure volume in a reference volume (e.g. cytoplasm) can be estimated by counting the ratio of points that fall on structure (Golgi component of interest) versus the points that fall on the reference volume (e.g. cytoplasm). Points are defined by the corners that lie between grid-lines or crosses arranged in a regular lattice pattern. **b** The packing density of surface in a reference volume is estimated using interactions between randomly positioned and oriented test lines and Golgi membranes. Sample position is randomised using SUR sampling and orientation randomised using the isector [embedding in a ball of gelatin, which is rolled randomly before slicing; Nyengaard and Gundersen 1992; isotropic uniform random (IUR) section]. Systematic arrays of test lines are superimposed on images and intersection counts (*I*) between line edges and Golgi membranes counted (*red arrowheads*). Surface density, *Sv* = *2I*/*L*, where line length *L* applied to the reference space (e.g. Golgi stack) is estimated by counting points over Golgi cisternae (*blue arrows*) multiplied by the line length associated with each point. **c** In the vertical section (VS) method, a section is cut in a vertical direction, orthogonal to a convenient/arbitrary horizontal plane such as the bottom of a culture dish. The section must have random placement and freedom of orientation around the vertical. Cycloid arcs aligned with the *vertical direction* represent a full distribution of isotropic lines in 3D space. Intersections (*I; red* *arrrowheads*) are counted and surface density, *Sv* = *2I*/*L*, where again, *L* is estimated by point counting (*blue arrows*) as described in (**b**). **d** Local vertical windows (LVWs). In IUR sections, the animal cell Golgi twists and turns in 3D making it difficult to identify and quantify regional elements (e.g. *cis, medial* and *trans*). Local vertical windows (LVWs) first “choose” stack profiles with membranes that present clear cisternal membrane profiles with minimal thickness. These represent Golgi stacks sectioned in a *cis–trans* direction. A line of best fit drawn “parallel” to Golgi cisternae membranes now traces a horizontal plane in 3D (*dashed line*) with the vertical in a *cis–trans* direction. A cycloid array is used to estimate the *Sv* of any membrane or substructure across this axis estimated using the same formula as above (*red arrowheads* indicate intersections between cycloids and cisternal membranes; point counts for estimating line length omitted for clarity). *Graph* in (**e**) compares estimates obtained using LVWs and isotropic line methods. For these data multiple Golgi stacks were generated in RK-13 cells using the drug nocodazole. Estimates obtained with the LVW method stabilized faster than for the isotropic lines method across a series of images. LVWs, *N* = 12 images and for isotropic line, *N* = 20. *Error bars* are standard error of the mean calculated for a ratio estimate according to Cochran (1977). Section orientation requirements: R-sections with random orientations; VS-sections with vertical orientation

**Fig. 4:**
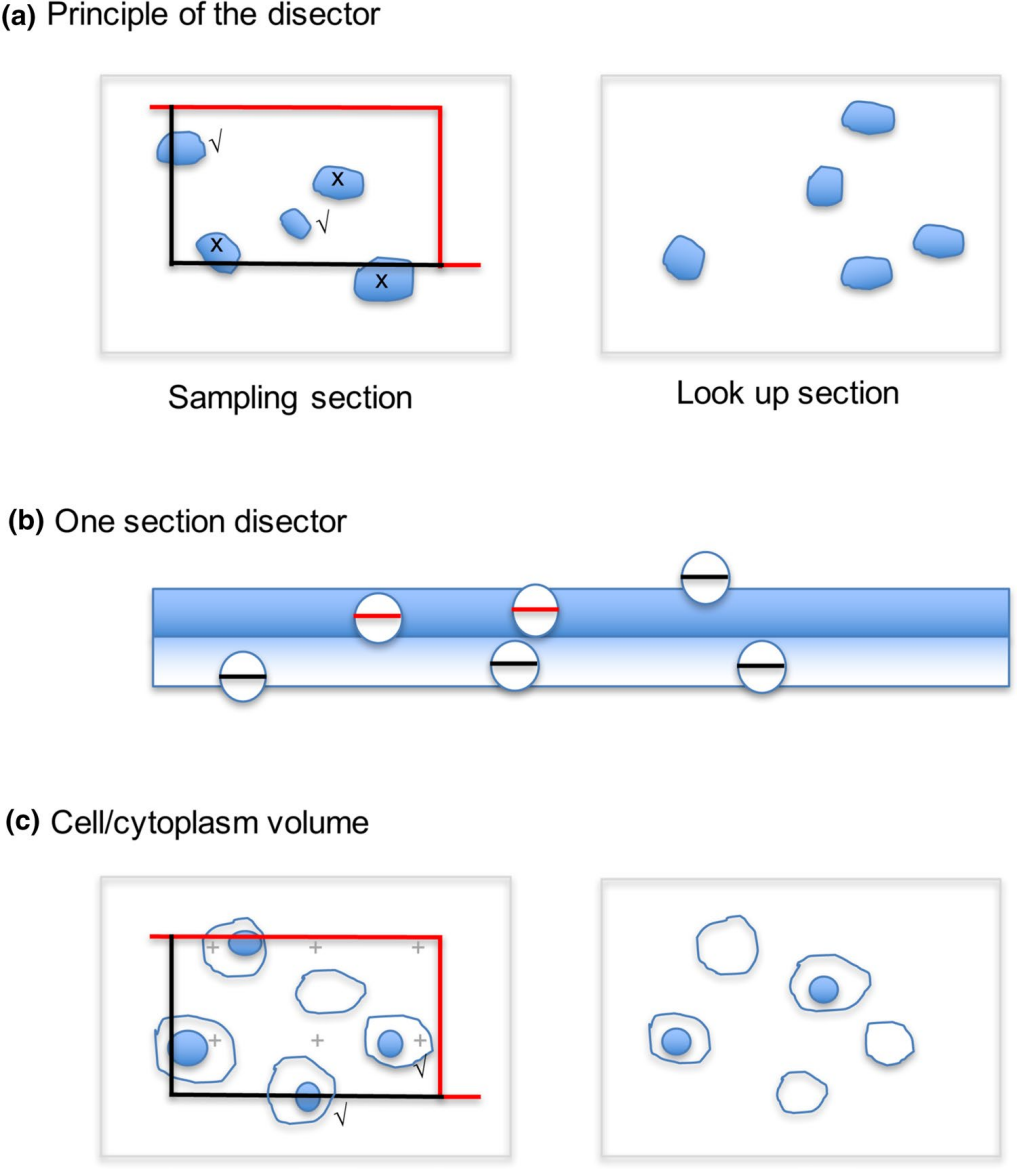
Single section analysis of number and reference volume. Number estimation requires a volume probe composed of two sections with known spacing (a disector). **a** The principle is to select particle profiles on one plane (sampling section) using an unbiased counting rule applied to a counting frame (quadrat). Particles are selected if enclosed either partially or completely within the frame and are not crossed by the forbidden line (*red*). Particles are counted only if they disappear in the lookup section (*Q* ^−^). **b** In conventional TEM, when the section thickness approaches the diameter of the structures (e.g. for 60-nm COPI vesicles), a single section can be used for counting (the workup checks that the fraction of equatorial profiles generated from one section (*red lines*) that cannot be identified as such in the next adjacent section). This approach is called a one-section disector. However, care must be exercised when using this approach with volume SEM methods. One problem occurs when SEM imaging comes from surface layer of a much thicker section, so that vesicles waists will be missed (e.g. Array-SEM or SBF-SEM). In this case increasing the imaging depth could solve this problem. Another possibility is that imaging and section thickness are very small compared to the vesicle size (e.g. with FIBSEM) so that the same vesicle waist may appear in multiple sections (thereby decreasing the fraction of vesicle profiles that disappear in adjacent sections). In this case it could be useful to assemble a projection image from a ministack of sections, which itself approximates in thickness to size of the vesicles in question. The numerical density of vesicles (or fenestrations for example) counted in disectors can be related to volume by performing point counting for the appropriate reference space found within the disector (not shown). A systematic lattice of points is applied to the sampling frame and the number hitting the reference space profile is recorded. The estimate of the volume of reference space inside the disector is *P* × *a* × *h*, where *P* is the sum of points, *a* is the area associated with one point on the lattice and *h* is the distance between the section planes used to make the disector. Numerical density is given by *Q* ^−^/ *P* × *a* × *h*. **c** The mean volume of a reference component or space (e.g. cell cytoplasm) estimated using a disector; (see **a**). Structures in the disector (ticks; *Q* ^−^) and points (*P)* over the component/space profiles are counted. Mean volume is given by: *P* × *a* × *h*/*Q* ^−^. Again the second section is prepared parallel to the first and distance between sections (*h*) is computed. Low-magnification SEM images are used for disector and point counting

**Fig. 5:**
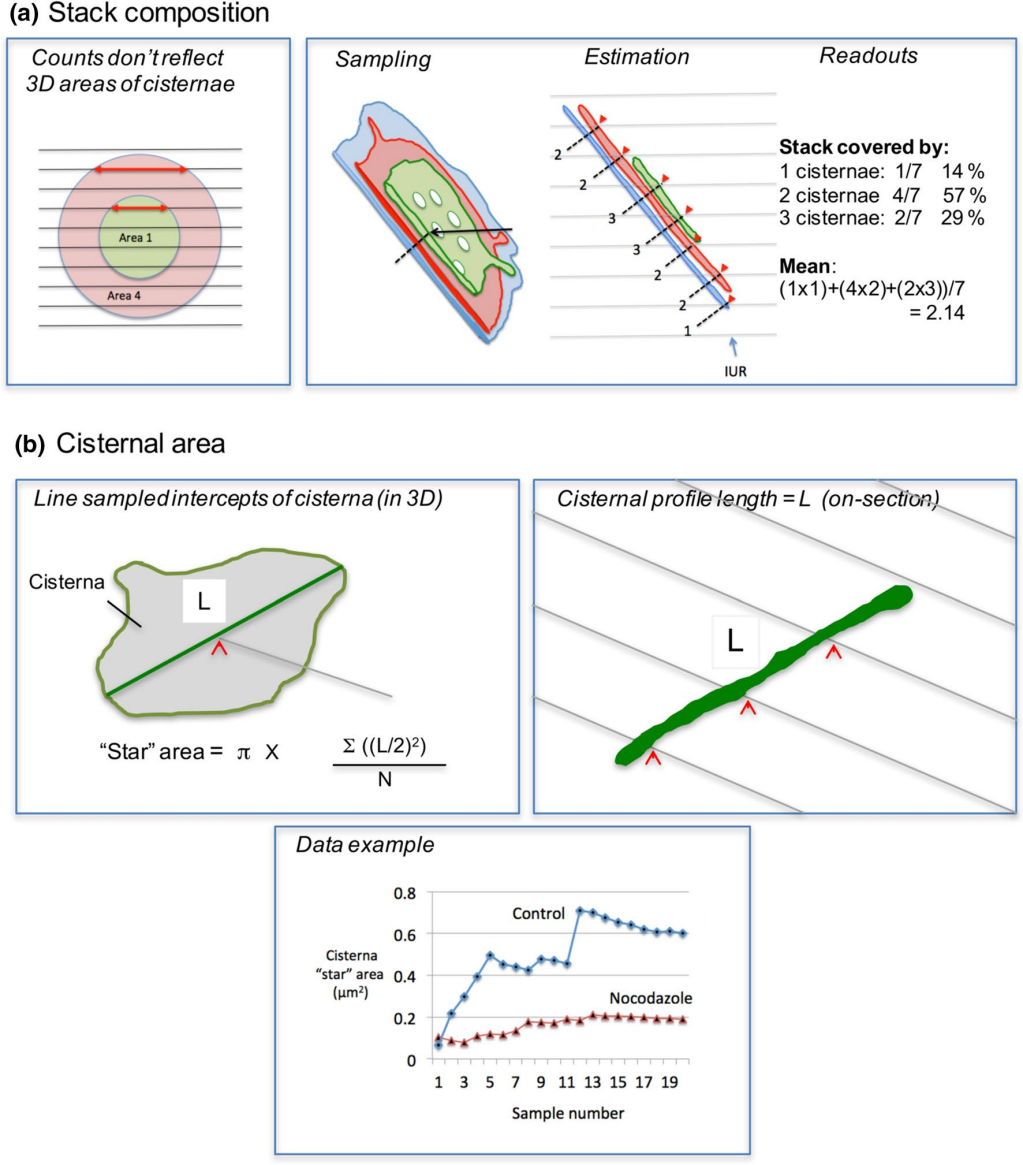
Single sections: specialised estimators. **a** Composition. Counts of cisternae in sections are a biased measure of relative 3D number (Lucocq and Hacker 2013). A randomly positioned stack of sections hits a larger (*red*) cisterna eight times and the smaller (*green*) cisterna four times reflecting the twofold greater height of the large cisterna. By contrast there is a fourfold difference between relative areas of the two cisternae in 3D. This is reported by relative profile length on the sections (*red arrows* mark the lengths of two sectioned profiles). The composition of the stack “ribbon” can be sensed using SUR test lines with random placement and isotropic uniform random (IUR) orientation. At each intercept the number of cisternae in the stack is counted in a direction orthogonal to the stack (*dashed lines*). Results are summed from multiple SUR images and provide estimates of proportions of the stack ribbon covered by 1,2,3, etc. cisternae. **b** Cisternal “spread” or “star” area. *Top left* Principle: the IUR *grey line* identifies a randomly positioned intercept (*red arrowhead*) on the cisternal surface (*grey fill*). The randomly oriented intercept (*green*) passes through the sampled point. The length of the intercept is used to compute the area of the surface from the equation. *Top right* in a real sample (randomised in orientation during embedding), random locations on a cisterna (sectioned profile in green) are located (*red arrowheads*) using a systematic array of test lines (*grey*) applied to a section. The length of cisternal profile represents *L* and is estimated for each random sampling “hit” using the same test system of lines. In the example for each random hit there are three line hits on the green cisterna profile. Each of these hit totals report an estimated cisternal length of *π*/*2* × *I* × *d* where *I* is the number of intersections (3) and *d* is the real spacing of the test lines (the grid must be randomised in orientation and position relative to the cisternal profile). Notice that this estimator of intercept (cisternal profile) length works for curvilinear profiles. Star area will reflect the degree of connectivity/extent of the cisternae in 3D. Bottom: star areas of Golgi cisternae in RK13 cells with and without fragmentation of the Golgi using the microtubule depolymerising agent nocodazole (sample number indicates the number of SUR images used for each cumulative estimate from this single illustrative experiment)

**Fig. 6:**
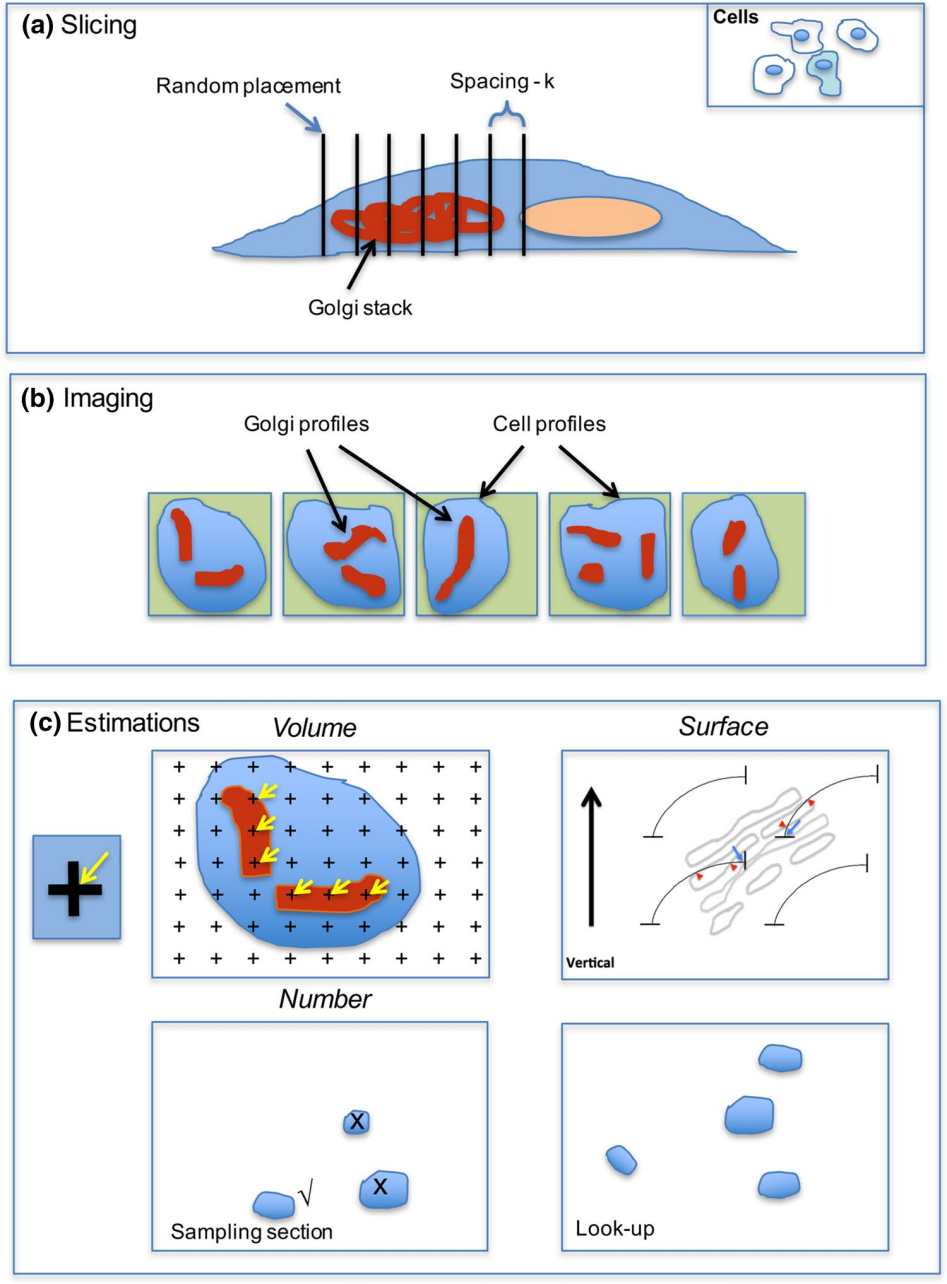
Parallel section stacks (Golgi subpopulations/individual cells). The cell(s) of interest is (are) identified (for example, using correlative light and electron microscopy (CLEM)) and prepared for SEM. **a** and **b** A stack of 5–10 parallel images is recorded spanning the entire Golgi organelle. The stack is positioned at random and each section evenly spaced by a known interval/section number. In FIBSEM the orientation of the sections is easily set as orthogonal to the horizontal plane of the cell culture dish (vertical section). **c** *Estimations* Volume of Golgi structures can be estimated using Cavalieri’s volume estimator *V* = Σ *A* × *k*, where *A* is the area of sectioned profiles summed over all section planes and *k* is the spacing of the images. Area *A* can be computed from *P* × *a*, where *P* is the sum of points (*black crosses*) hitting the profiles on all the stack images and *a*, is the area associated with each point on the grid lattice (applied SUR). The estimator is unbiased if section planes used are infinitely thin with respect to the object of interest. Corrections may need to be applied when slice thickness is substantial compared with the spacing *k* (Howard and Reed 2005). When sections are vertical, the surface density of Golgi component membranes can be estimated using a cycloid arc test system applied along the vertical direction (SURS pattern; see text and Fig. 3). The total surface is obtained by multiplying *Sv* by reference volume obtained using the Cavalieri estimator. Number can be estimated using at least two sections in close proximity or adjacent to each other in the stack (*bottom left* and *right*). Structures that produce profiles in one section, but not the next (√) have edges between the sections and, therefore, are counted (disector principle; Sterio 1984; Gundersen 1986). The sections need to be spaced at 1/3 to 1/4 of the diameter of the structures counted to make structures easy to follow. For convex objects such as vesicles, counting is straightforward because each object has a single edge (regardless of its size). One of the sections can belong to the section stack used for volume or surface estimation. The total number of structures in the Golgi is estimated from the number of disappearing profiles multiplied by 1/(fraction of sections used for counting) (Gundersen 1986; Lucocq et al. 1989; Smythe et al. 1989). For example, if COPI vesicles are counted using ten pairs of adjacent 20-nm sections through a 5-µm Golgi and the distance between the pairs is 50 sections then the estimate of COPI number is vesicle edge counts (Q−) × 50/2 (the denominator is 2 because counting can be done in both directions to improve efficiency). If there are 500–1000 COPI vesicles then 20–40 vesicles will be counted for each cell. If the section thickness approaches the size of the vesicles (as is the case of conventional 50 nm resin sections and 60–70 nm COPI vesicles) a majority of vesicle equators detected in the sampling section may be absent in the lookup section. Under these conditions a single section disector (Lucocq et al. 1989; Nyengaard and Gundersen 2006) is established and comparison with the lookup section is not necessary, speeding up the analysis. Care must be exercised when designing single section disectors for use with the surface imaging used in volume SEM (see Fig. 4b and text for details). Numerical density can be computed by combining disector counts with point counting or surface estimators. Another possible readout is to relate the number of one structure to another using the ratio of counts (Gundersen 1986)

The second element in stereological design is application of estimators for sensing 3D quantities from 2D profiles in the SEM or TEM image. The use of geometrical probes (Figs. 2, 3, 4) is carefully designed to allow unbiased and efficient estimates of quantities such as volume, surface, length or number. The probes (points, lines, planes) are applied to images on which counts of interactions with the compartments of interest can be converted into relevant structural quantities. A biological specimen (organ, tissue, cell, cell compartment) is one of a number of independent sampling items or replication units in a set. Remarkably, SUR sampling of stereological estimators through the specimen of interest is an efficient strategy for quantitative analysis. Often, only 100–200 counts of chance probe–structure interaction are required per specimen to achieve acceptable levels of estimation precision for comparing different (e.g. control and experimentally-treated) specimen sets (Howard and Reed 2005; Mayhew and Lucocq 2015).

In the following, we discuss how (1) stereology of single SEM slices can be used to sense accurately global changes in a Golgi population and (2) stereology applied to local arrays of parallel sections can sense 3D parameters in individual Golgi organelles. The “parallel section stack” approach greatly increases the number of single cells that can be analysed allowing the sensing of both inter-cell variability and the population parameters of interest—a significant improvement in efficiency compared to the serial reconstruction from exhaustive sectioning. Importantly, the application of parallel section stacks will allow the study of Golgi in rare subpopulations (Fig. 2, right; Narayan and Subramaniam 2015; Russell et al. 2017) such as those generated by correlative LM and EM (CLEM; Flottmann et al. 2013), where individually treated cells (or groups of them) are first identified using LM before being relocated and examined in EM.

## Single section/image sensing: sampling and estimators

Single, wide-ranging sections/images can be prepared by SBF-SEM, array-SEM and also TEM. The size of the section is set by the width of the diamond knife, and can range across mm size cell pellets or tissue blocks. FIBSEM images come from a more restricted block face, which makes them less suited to single slice/image analysis because they are set locally in the sample.

To preserve links with the “ground truth” parameters in the 3D sample, the pellets/blocks, section and imaging should be part of a random sampling scheme illustrated in Fig. 2. Typically, high magnification images are collected in an SUR array to which geometrical feature probes are applied as randomly placed SUR test arrays. Stereology on a single slice/image efficiently primarily senses densities (concentrations) of parameters such as compartment volume, surface or number inside a reference volume (e.g. volume, surface or number of vesicles in a Golgi complex or cell volume). For volume density (fraction) analysis, systematic arrays of test point probes are applied to the images (Fig. 3a) and the test points “landing” on a Golgi structure and on a suitable reference volume (say, the cell) are counted. The fraction of points falling on the Golgi structure is then an unbiased estimate of its volume fraction within the cell.

Similarly, packing density of surface in reference volume (surface density) is estimated using test line intersection counting (see Fig. 3b). In this case, randomly oriented (isotropic) encounters between the sample and probe are required. This can be achieved by embedding the sample in a ball of gelatine, which is rolled prior to embedding and slicing and imaging (isector, Nyengaard and Gundersen 1992).

Another approach to surface estimation is the vertical section method, which confers isotropy when sine-weighted or cycloid test lines are used (Fig. 3c; Baddeley et al. 1986). Here, the section is prepared along (and randomly rotated around) a vertical axis which itself lies at right angles to an arbitrarily chosen horizontal plane. By placing a systematic array of cycloid arcs or sine-weighted test lines over a micrograph, the surface density can be estimated unbiasedly. The horizontal plane can be chosen by the investigator and could conveniently be represented by a tissue plane or the bottom of a cell culture dish.

The Golgi can be conveniently analysed across the *cis–trans* axis using a specialised application of the vertical section principle. This approach uses local images of Golgi stacks recorded from sections that are already randomly positioned and randomly oriented (not vertical). The investigator now searches for Golgi stacks where the section passes orthogonal to the membranes ignoring tangentially sectioned profiles. This creates a so-called local vertical window (LVW; Fig. 3d), on which cycloid arcs can be placed according to the vertical (now *cis–trans*) direction. Figure 3e compares estimates for Golgi stack membrane surface density obtained using LVWs and the traditional line intersection method on the same sample. LVWs have the advantage of preserving orientation (Bruel and Nyengaard 2005) along the *cis*–*trans* axis of the Golgi stack and could be used for analysing stack composition or regional differences in the surface areas of vesicle, tubule or cisternal membrane pools (see below).

In most cases, number density cannot be sensed on single sections. Rather a volume probe composed of two parallel sections is required (Sterio 1984; Gundersen 1986; Lucocq and Hacker 2013). One of these sections is used to sample the profiles unbiasedly and the other is used to detect the profiles that disappear (see Fig. 4a). The disappearing profiles then have edges situated in the volume between the slices and are counted. The volume probe formed by the two parallel sections is called a disector (Sterio 1984; Gundersen 1986).

A special case occurs in conventional TEM when structures, such as vesicles, and the sections used are comparable in size (e.g. Golgi vesicles measure 60–70 nm and TEM sections, 50–100 nm; Lucocq et al. 1989; Nyengaard and Gundersen 1992). Now, conveniently, a substantial fraction of vesicle equators will appear in one section but not the next—effectively making a one-section disector (Fig. 4b). Knowledge of the average section thickness, combined with point counting for estimating the reference volume, provides an estimate of numerical density. The procedure involves first checking in two parallel sections the probability that vesicle structures under investigation will “disappear” in the look-up section and assumes vesicle size is unchanging across different analysis conditions. It is important to note, however, that when the imaging depth of volume SEM techniques is substantially less than the physical section thickness, the use of a one-section disector can result in the loss of Golgi vesicle waists leading to bias in the estimates (see Fig. 4b legend for details).

Counting vesicles unbiasedly is known to be important in cell biology (Smythe et al. 1989). There are thousands of vesicles present in the Golgi region (Marsh et al. 2001) and they belong to two principal vesicle populations with cytoplasmic coats composed of coat protein 1 (COPI) or clathrin (Faini et al. 2012, 2013; Martínez-Alonso et al. 2013; Jackson 2014). Quantitative studies of COPI vesicles are limited to the LM level (e.g. Pepperkok et al. 2000) or to small samples examined by tomography, while clathrin-coated vesicles have been quantified at the plasma membrane (Smythe et al. 1989).

With the stereological estimators discussed above, it is important to be conscious of the potential pitfalls. The density of volume, surface or number of Golgi structures are especially useful in screening procedures, but densities are sensitive to the size of the reference volume (reference trap; Braendgaard and Gundersen 1986; Howard and Reed 2005). For instance, a decrease in the reference volume can increase the density of a given compartment without any real change in its absolute quantity. A quick aid to interpretation is to use two sections with known spacing in a disector (imaged conveniently in the SEM (or indeed in TEM) at low magnification) to estimate the reference space volume (such as the cytoplasm) per cell as illustrated in Fig. 4c.

### More specialised estimators of Golgi complex composition and size

The composition of the Golgi membrane structures is of interest in studies of assembly/disassembly during the cell cycle, or in response to pharmacological agents or changes in gene expression (e.g. Klausner et al. 1992; Storrie et al. 2012). Stereology can be used to estimate the fraction of membrane in Golgi cisternae of different types (e.g. *cis*/*trans)*, or the fraction of membrane in say vesicles versus cisternae. In this case, imaging windows from single randomly positioned and oriented slices/images are probed using test lines. The lines are applied to images as SUR arrays (Fig. 3b) or conveniently as a single scanning line directly on the camera image at the electron microscope (Lucocq 2003, 2008) for rapid results. Line intersects report on proportion of surface areas of relevant membranes in 3D (Griffiths et al. 1989; see; Baddeley and Jensen 2004). As mentioned above, cycloid test lines placed on images of vertical sections can also be employed for this purpose.

Stack composition can also be estimated by first sampling the Golgi ribbon surface (face) using line probes, and counting cisternal profiles orthogonal to the sampling point (Fig. 5a; Lovelock and Lucocq 1998). This type of analysis avoids the bias produced when simple relative counts of cisternal profiles are used (see Fig. 5a). This estimate requires random orientation of the orthogonals in 3D and is, therefore, not suited to the vertical section approach. In this case stack composition can be analysed by estimating the relative sizes of the cisternae using relative intersection counts on vertical sections.

Individual Golgi cisternae are complex structures that are extensively interconnected in the centrosomal region of the animal cell producing plate-like or ribbon-like structures in 3D. While single slices/images cannot be used to estimate the extent of the cisterna/ribbon directly, a quantity known as “star area” is accessible. Star area estimates the “visible” area as “viewed” from a randomly selected location on a Golgi cisterna. Star area is estimated using intercepts across a planar surface (Gundersen et al. 1988) and the intercept is generated by the section as it passes across the cisterna in 3D (simply then the length of the cisternal profile on the section; Fig. 5b). Star area is sensitive to the connectedness of the Golgi ribbon and/or the extent of the cisterna/ribbon in 3D. Data presented in Fig. 5b are based on estimates of star area with the aim of following fragmentation of the Golgi after treatment with the microtubule depolymerizing drug nocodazole.

The arithmetic mean thickness of the Golgi stack can be computed from the mean of measurements across the structure from random locations multiplied by *π*/4. An alternative is the harmonic mean thickness (Knust et al. 2009; Jensen et al. 1979), which is more resistant to effects of numerically large outliers [in this case these are generated when cisternae become sectioned at oblique angles (Hirose et al. 1982)]. Average thickness of individual cisternae can also be estimated using the packing density of cisternal membranes. Here the cisterna is modelled as a flat plate in which thickness approximates to *2*/*Sv* [from rearrangement of *Sv* = *2*× *area*/*volume* = *2* × *(a*/(*t* × *a*)] where *a* is the area of one aspect of the cistern, *t* is the thickness and *v* represents the cisternal volume as reference space (ignoring the rim membrane; not shown). Surface density estimation is described above.

## Stereology on parallel section stacks for quicker estimates on individual Golgi

This parallel section stack approach has been studied extensively for estimating volume and densities in single biological items such as organisms, organs, cells and organelles (Cruz-Orive and Myking 1981; Gundersen and Jensen 1987). These studies suggest that volume and also component densities are estimated with reasonable precision using a systematic array of 5–10 parallel and equally spaced sections. For unbiased results the parallel section stack is positioned at random and spans the entire organelle/compartment of interest (Fig. 2).

A key point here is that while parallel section stacks are quite laborious to prepare manually by conventional means (see Lucocq et al. 1989), the newer volume-SEM methods provide arrays of automated sections and/or images from which the parallel sections can be selected (Figs. 2, 6; Kizilyaprak et al. 2014; Peddie and Collinson 2014; Hughes et al. 2014). The section stack approach is suitable for SBF-SEM and array SEM imaging and also FIBSEM while in SBF-SEM/array SEM there is freedom to choose the orientation of imaging (random section approach). In FIBSEM the orientation is less flexible as most studies are performed orthogonal to the horizontal plane in the microscope/specimen, which restricts the use of some estimators (see below). It is worth noting, however, that orientation constraints of FIBSEM imaging can be overcome by virtual sectioning using in silico reconstructions, or by re-mounting or re-embedding of samples prior to further imaging. However, these methods do require greater data storage and an increase in sample handling time, respectively.

Stereological estimators are applied to each of the parallel sections and integrated estimations of volume, surface and number densities and also absolute quantities—can all be made from the same stack of sections (Fig. 6). This approach avoids the “reference trap” mentioned above. In the case of volume-SEM, selecting a small subset of the exhaustive serial sections/images reduces overall workload and time for analysing individual cells by orders of magnitude, allowing multiple cells to be analysed. As an example, for SBF-SEM, if a typical Golgi apparatus was sectioned over five microns using 200, 25-nm sections the 5 parallel sections in the stack used for stereology would comprise 2.5% of the total. In the case of FIBSEM there could be 1000, 5-nm sections, and the parallel stack sections would make up 0.5%—a substantial reduction in the need for data processing, retrieval and storage.

### Volume

The volume of a cell or cell component can be estimated using section stacks using the principle based on the work of Bonaventura Cavalieri (Howard and Reed 2005; Fig. 6a–c). The volume could be an individual component (e.g. Golgi stack) or it could be a reference volume used to convert organelle surface, volume or number densities into absolute amounts (Weibel 1979; Howard and Reed 2005; Lucocq 2008). The Cavalieri principle uses a randomly placed stack of sections, with constant known average spacing, spanning the entire volume/space of interest (an application of SUR sampling; Fig. 6c). The volume estimate is then obtained from the total area of structure profiles on the slices, multiplied by the distance between sections. The number of sections required for a precision of about 5% is usually between five and eight (Gundersen and Jensen 1987). Conveniently, Cavalieri estimates allow the orientation of the sections to be fixed arbitrarily, according to the constraints of good imaging or the physical attributes of the system. Thus, certain orientations of the Golgi may allow better recognition of stack structure or the investigator might be forced to restrict the orientation to a vertical direction across a cell monolayer, as is possible with FIBSEM imaging. Sections with arbitrary orientations can also be used for estimating number but may not be used for 3D surface estimations, which, instead, require randomly oriented or vertical sections to be prepared.

### Membrane surface

Sections stacks can be used to estimate the membrane surface in Golgi compartments by employing line intersection methods similar to those described for single sections. The surface density of Golgi cisternal membranes, tubules or vesicles is first estimated inside a chosen reference space (e.g. the cell; Fig. 6b, c). The total surface of membrane can then be computed directly from the product of surface density and reference volume (obtained using the Cavalieri estimator).

As with single section analysis (described above) interactions between the sample and test lines must be oriented randomly. One way is to enclose the sample in gelatine and rotate before sectioning and apply an SUR array of lines for surface density estimation. However, because the orientation of each section in the stack tends to be consistent, the results will be sensitive to any preferred orientations (anisotropy) of the (Golgi) membranes in different cells. Although unbiased at the population level, this effect will increase inter-cell/Golgi variation and decrease precision. An alternative approach is to generate isotropic lines in space by combined use of vertical sections and cycloid arrays (Figs. 3c, 6c; Baddeley et al. 1986). The vertical axis lies at right angles to an arbitrarily chosen horizontal plane such as the bottom surface of a cell culture dish. FIBSEM is ideally suited to the application of the vertical section method because the plane of imaging is conveniently orthogonal to the base of the specimen (horizontal plane). Notice also that intersection counts using vertical sections can provide data on membrane composition although more specialised estimators for stack composition/cisternal size (Fig. 5) require randomly oriented sections.

### Number

Section stacks are a powerful way of estimating the total number of particulate structures (e.g. Golgi vesicles). The key is to count particles using a disector, i.e. two parallel sections (images) at each position in the systematic section array. One of these two sections (or images) can belong to the parallel section stack and the other is imaged at a distance equivalent to one-third to one-quarter of particle height along the axis of sectioning (Sterio 1984; Gundersen 1986). The number of profiles that disappear from one section to the next provides an unbiased estimate of particle number in the sampled disector volume (Figs. 4, 6). Efficiency can be improved by counting in both directions in the section stack because each direction will ‘capture’ different structures.

As described above for single slice analysis, a special case called the one section disector arises when the particles being counted are similar in size to the sections used to investigate them (e.g. when Golgi vesicles of 60–70 nm are sectioned using conventional microtomes at 50–100 nm in TEM; Fig. 4; Lucocq et al. 1989). Here a disector formed from two adjacent sections is prepared and the number of disappearing profiles is counted (Fig. 6). If the majority of the vesicle profiles (say 95/100 of all counted vesicles) then disappear, the disector count approximates to the structure counts in a single section (see Fig. 4). Number is then estimated by multiplying by the number of sections found in the stack interval. As already discussed, in the case of SEM imaging, care must be taken to ensure the imaging depth in the sections is comparable to the physical section thickness used, otherwise vesicle waists will be lost leading to underestimates of vesicle number (see Fig. 4b legend for details).

A specific problem arises when data from individual cells are required and the cells of interest form part of a wider cell population that is distributed in 3D space. Here selection of cells according to their number (cardinality), rather than some other characteristic such as size, is important. This can be achieved using a disector that selects the cells at one end of the parallel section stack used for stereological estimations (Lucocq et al. 1989). At low magnification the end section of the stack is used as the look-up section and the last but one as the sampling section (not shown). Cell or nuclei profiles that are selected by the counting frame on the sampling section and that disappear in the look up are then quantified in the rest of the section stack using the stereological estimators described above.

### Application of specialised estimators to parallel section stacks

As we have described above, a set of specialised estimators for cisterna and stack thickness, stack composition and cisternal area can be applied to randomly oriented SEM sections. These can also be applied to randomly oriented section stacks with the caveat that preferred orientation of Golgi membranes within individual cells may increase the cell-to-cell variation of the estimates. In the restricted orientations provided by vertical sections (commonly prepared by FIBSEM), star area estimation is problematic while stack composition is better estimated on vertical sections with estimations of relative membrane surface of the different cisternae obtained using counts of cycloid arc intersections (see above).

## Systematic errors

Objects of small size are subject to systematic errors (biases), especially when slice thickness approximates the size of target structures (e.g. with Golgi vesicles/tubules and conventional 50 nm sections). Errors are particularly evident when estimating volume or surface. One bias reduces estimates of vesicle volume and surface and stems from unclear images of the structure periphery—an effect that is also known as “lost caps”. A second bias increases estimates of volume and surface and stems from overprojection of structures inside the slice into the image (the Holmes effect; Weibel 1979). Model-based estimates of the bias for surface and volume densities of Golgi vesicles or tubules can not only be calculated (Weibel and Paumgartner 1979) but can also be measured directly using EM tomographic sets that use much thinner sections (Vanhecke et al. 2007). Corrections for overprojection in Cavalieri estimates are also available (Howard and Reed 2005). Thankfully the imaging thickness in volume SEM is reduced because the signal comes from the surface (5 nm or less), making these errors less significant. For higher throughput analysis, systematic errors may be accepted if the size of small structures remains constant under different conditions. In such cases, the relative biases will be the same in different treatment groups and estimated quantities will retain their comparative worth (Mayhew and Lucocq 2015).

One important factor for any type of quantitative estimation in EM (including volume) is rigorous application of criteria for compartment or structure identification (in this case the Golgi). Thus, boundaries of interesting structural profiles must be reliably identifiable and map onto and delineate the 3D objects that generated them. In short, one cannot measure that which one cannot identify. For example, in EM sections/images, cisternae can be identified as membrane-bound profiles of specified size and asymmetry that are not coated by ribosomes (e.g. a thickness of 60 nm and a long axis that exceeds the short axis by threefold). A limited serial section analysis offers a convenient way to establish the reliability of the chosen criteria for identifying cisternal structures in 3D. For high throughput, errors in identification may increase the bias but this may be deemed acceptable if relative biases are similar in different study groups. Automated identification or recognition is a potential growth area for high-throughput TEM (e.g. Kreshuk et al. 2014; Fordyce et al. 2014; Higaki et al. 2015). While most quantification is currently carried out using “manual” segmentation, automated machine recognition/learning is improving and some algorithms now exhibit performance that approximates the human brain-eye combination (not discussed further here).

Another error of importance can occur at the micrograph edge. Golgi structure profiles situated here may have critical features needed to identify them when they lie partly outside the boundary of the image. This effect can be avoided by surrounding the quadrat used for estimations by a guard area, which is big enough to identify all possible structures regardless of size and shape. We recommend the routine use of a guard area. In the case of section stacks, when the whole Golgi is of interest and has its own natural boundaries a guard area is not required.

## Final comments

**Fig. 7:**
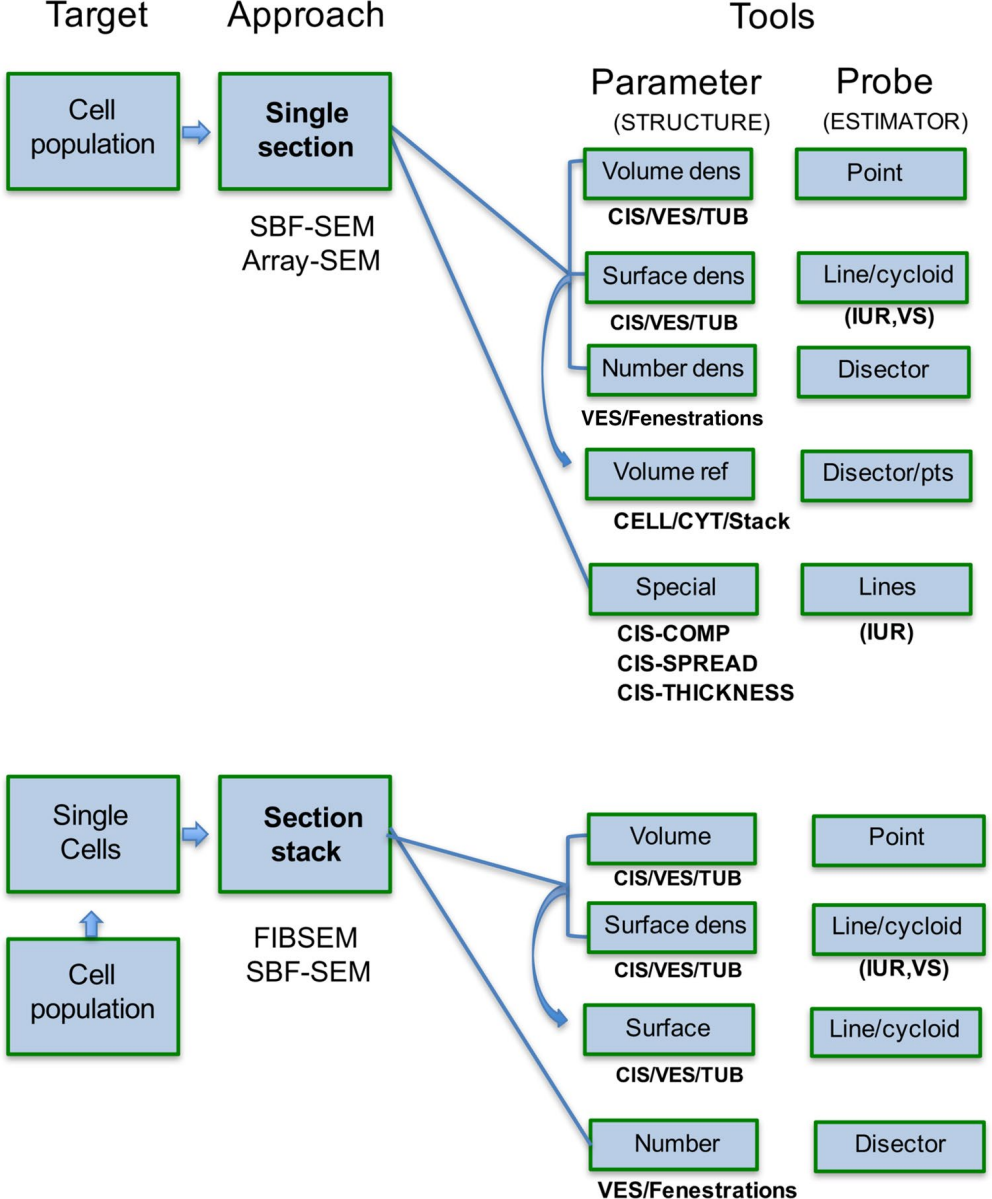
Experimental design for stereological estimation on single slices and parallel section series in volume-SEM. Two experimental pathways are outlined here, using either single sections for investigating the parameters of Golgi populations, or randomly placed parallel section stacks for analysis of subpopulations/individual Golgi. The single section approach is more suited to SBF-SEM and array-SEM, because they produce larger section areas than FIBSEM. Here the primary accessible parameters are densities, which may be converted to amounts using estimates of the reference volume (*curved arrow*). In the case of parallel section stacks, absolute volume and number are directly accessible but surface must be determined as a density first before combining with the volume estimate to derive membrane surface (*curved arrow*). In either approach volume and number can be determined on sections that are arbitrary, randomly oriented or vertically oriented. Surface density and specialised estimators require isotropic uniform random (IUR) sections or vertical sections (VS; details are given in the text). *CIS* cisternae, *TUB* tubules, *VES* vesicles, *CYT* cytoplasm, *Fenestr* fenestrations

The strength of newer volume-SEM technologies resides in their ability to produce large amounts of serial sections and/or serial images. But the challenge is to process the information in a way that connects with the 3D reality of organelles (such as the Golgi) at both specimen and population levels, without engaging in massive amounts of work. A potential weakness of volume-SEM occurs when the investigator is drawn into analyzing large amounts of 3D information at a restricted number of cell locations. This not only takes time and produces digital waste but also requires substantial in silico storage. Sampling-based stereology offers an opportunity of sensing the Golgi population more widely than with exhaustive sectioning but retains enough information about the individual items and their parent population to provide estimates with satisfactory levels of precision. This is particularly important when EM is used for screening purposes, as in CLEM. With judicious selection of target parameters (volume, surface or number) combined with careful study design the “threat” of large data sets can then be averted, leading to efficient and accurate sensing of the “real” Golgi in 3D space (see Fig. 7).
